# Effectiveness and Safety of Chinese Herbal Injections Combined with Fluoropyrimidine and Oxaliplatin-based Chemotherapy for Advanced Colorectal Cancer: A Systematic Review and Meta-analysis of 63 Randomized Controlled Trials

**DOI:** 10.7150/jca.60895

**Published:** 2021-10-25

**Authors:** Shuo Wang, Xueqian Wang, Tong Zhou, Shuaihang Hu, Peiyu Tian, Zheng Li, Yuxiao Li, Jun Dong, Yuerong Gui, Dandan Wang, Ying Zhang, Wei Hou

**Affiliations:** 1 Department of Oncology, China Academy of Chinese Medical Sciences Guang'anmen Hospital, Beijing, China.; 2 Beijing University of Chinese Medicine, Beijing, China.

**Keywords:** advanced colorectal cancer, Chinese herbal injections, effectiveness, randomized controlled trial, systematic review

## Abstract

**Purpose:** To investigate effectiveness and safety of Chinese herbal injections (CHIs) in conjunction with fluoropyrimidine and oxaliplatin-based chemotherapy (FOBC) for advanced colorectal cancer (CRC).

**Methods:** A comprehensive search was conducted in 7 electronic databases for related randomized controlled trials (RCTs) from inception to April 30, 2021. The quality of each trial was assessed according to the Cochrane Handbook for Systematic Reviews of Interventions, the differences in effectiveness and safety outcomes between two groups were evaluated, and the results were expressed as the risk ratios (RRs) and 95% confidence interval (CI). Subgroup analyses were performed according to the types of CHIs, and Review Manager 5 was used to statistically analyze the outcomes.

**Results:** 63 studies involving 9 CHIs and 4733 patients were included in this review. The meta-analysis results suggested that compared with FOBC therapy, CHIs plus FOBC therapy showed significant improvements in objective response rate (ORR) (RR=1.34, 95% CI: 1.27-1.42, *P*<0.00001), disease control rate (DCR) (RR=1.09, 95%CI: 1.06-1.11, *P*<0.00001), 1-year survival rate (RR=2.27, 95% CI: 1.23-4.18,* P*=0.009) and quality of life (QoL) (RR=1.21, 95% CI: 1.14-1.28, *P*<0.00001), and decreases in the incidence of chemotherapy-induced leukopenia (RR=0.64, 95% CI: 0.50-0.82, *P*<0.0005), nausea and vomiting (RR=0.65, 95% CI: 0.51-0.83, *P*=0.0005) and diarrhea (RR=0.34, 95% CI: 0.20-0.58, *P*<0.0001).

**Conclusion:** From the evidence available, CHIs could increase ORR, DCR and 1-year survival rate, improve QoL and relieve chemotherapy-induced leukopenia, nausea and vomiting and diarrhea when combined with FOBC in advanced CRC treatment, Nevertheless, on account of the limitations, more rigorous RCTs with high-quality methodology were needed to further confirm the results.

## Introduction

Colorectal cancer (CRC), the third most common cancer worldwide, is a serious threat to people's health and life. CRC is the second leading cause of death among cancers, with an estimated 935,173 deaths, counting 9.4% of all cancer deaths 1. Although surgery remains the primary treatment for CRC, 50% of patients recur or metastasize after radical resection. More than 25% of the patients confirmed in its advanced stage 2, with the overall 5-year survival rate ranges from 10 to 18%. Fluoropyrimidines and oxaliplatin-based chemotherapy (FOBC) is the first-line treatment for patients with advanced CRC, some cases can benefit from FOBC to improve survival as well as locoregional control 3. However, studies have demonstrated that it often accompanied by adverse reaction, further leading a reduced quality of life.

In Asia, Traditional Chinese Medicine (TCM) is a considerable adjuvant treatment for advanced CRC in combination with chemotherapy, and has been shown to increase effectiveness and reduce side effects. Chinese herbal injection (CHI), prepared by extracting and purifying effective ingredients from Chinese herbal medicines, is an important part of TCM 4. It breaks the limitations of the traditional delivery way of Chinese herbal medicines via oral administration, but intravenous injection instead, thus has the advantages of high bioavailability, high blood concentration, rapid action and no digestive tract absorption process. Many researches indicated that it has obvious advantages in improving short-term effectiveness, enhancing life quality and reducing chemotherapy-related toxicity. However, previous studies commonly focused on an individual CHI, while the types of CHIs are various, and the effectiveness and safety of all potential CHIs still remains inconclusive 5. Thus, a systematic review was designed to fill this knowledge gap by quantitatively synthesizing the evidence. The aim was to evaluate the effectiveness and safety of all potential CHIs for treating advanced CRC and to provide help for clinical medication in the future.

## Materials and Methods

This systematic review and meta-analysis was developed according to the Preferred Reporting Items for Systematic Reviews and Meta-Analysis (PRISMA) guidelines, and has been registered through International Platform of Registered Systematic Review and Meta-analysis Protocols (INPLASY) as INPLASY2020100050. The complete study protocol was previously published 6. Ethical approval was not required as all the research materials were published studies.

### Eligibility criteria

Only randomized controlled trials (RCTs) were selected and assessed for inclusion based on the following eligibility criteria: (1) Types of Participants: Patients were cytologically or pathologically confirmed cases of CRC and belong to Stage Ⅲ or Ⅳ according to American Joint Committee on Cancer Staging System (8^th^ edition) or mentioned “advanced”. (2) Types of Interventions: Control groups received FOBC that contained fluoropyrimidine and oxaliplatin, and the fluoropyrimidine drugs include 5-fluorouracil (5-FU) and Capecitabine. Treatment groups received CHIs plus FOBC therapy. In each trial, the FOBC regimen was eligible and the same in both treatment and control group. CHIs were given intravenously. (3) Types of Outcomes: The primary outcomes were objective response rate (ORR) and disease control rate (DCR). According to World Health Organization (WHO) 7 guidelines for solid tumor responses or Response Evaluation Criteria in Solid Tumors (RECIST) 8, the tumor responses were evaluated as complete response (CR), partial response (PR), stable disease (SD) and progressive disease (PD). ORR refers to the proportion of patients with CR plus PR. DCR calculated as the proportion of patients with CR plus PR plus SD. The secondary outcomes were progression-free survival (PFS), survival rate, quality of life (QoL) and safety outcomes. PFS defined as the time from study entry to relapse or death. Survival rate referred to the proportion of participants alive at the beginning of a time interval who survive to the end of the interval 6. Improvement of QoL was considered when Karnofsky performance scale (KPS) score increased, or decreased no more than 10 scores after treatment.^14^. Safety outcomes covered the incidence of grade 2 or greater leukopenia, diarrhea, and nausea and vomiting, measured by Standard Classification of WHO or National Cancer Institute Common Terminology Criteria for Adverse Events (NCI-CTCAE). The included studies should reported at least one of the above outcomes of interest.

### Information Sources

A comprehensive search was conducted from inception to April 30, 2021 in 7 electronic medical databases, including PubMed, EMBASE, Cochrane, China National Knowledge Infrastructure (CNKI), Wanfang Data, VIP Database for Chinese Technical Periodicals (VIP) and SinoMed.

### Search Strategy

To identify all potential relevant publications, search terms were constructed for two domains: (1) colorectal cancer, (2) CHIs. The terms used for colorectal cancer contained: “Colorectal Neoplasms[MeSH]”, “Colonic Neoplasms[MeSH]”, “Rectal Neoplasms[MeSH]”, “colorectal cancer”, “colorectal neoplasm”, “colorectal carcinoma”, “colorectal tumor”, etc. The following terms were used for CHIs: “Chinese herbal injection”, “injection of TCM” and certain CHIs such as “Shenqifuzheng”, “Kanglaite”, “Fufangkushen”, “Compound Kushen”, “Cinobufacini”, “Xiaoaiping”, “Elemene”, “Lentinan”, “javanica oil emulsio”, “kangai”, “Astragalus”, “Shenfu”, “Shenmai” and multiple synonyms for each term. More specific search strategies were showed on **File S1**.

### Study Selection

2 independent reviewers (J Dong and YR Gui) screened all the relevant articles on the basis of titles and abstracts. The full texts were scanned for further elimination based on the eligibility criteria. All disagreements were resolved by consensus. All relevant articles were managed in NOTEEXPRESS software.

### Data extraction

2 reviewers (T Zhou and SH Hu) completed the data extraction in Excel software independently, and the following items were extracted: general information including author, year, publication, sample size, detailed information of participants, intervention measures and outcomes. The disagreements between the 2 reviewers were settled by S Wang and Y Zhang.

### Risk of bias and quality assessment

We evaluated methodological quality of the included articles according to “Risk of Bias Assessment Tool” of the Cochrane Handbook for Randomized Controlled Trials 9. The risk of bias was evaluated in 7 items including random sequence generation, allocation concealment, blinding of participants and personnel, incomplete outcome data, selective reporting, and other sources of bias, and finally evaluated as “low risk,” “unclear risk,” or “high risk” 10. PY Tian and Z Li assessed the studies' quality independently, any differences were decided by XQ Wang and W Hou. The Grading of Recommendations, Assessment, Development and Evaluation (GRADE) system was used to grade the quality of evidence 11.

### Summary measures and data synthesis

Review Manager 5.3 was used to conduct statistical analyses. Risk ratios (RRs) were used to evaluate effectiveness and safety for dichotomous outcomes with 95% confidence intervals (CI). P values <0.05 were considered to indicate statistical significance. Subgroup analyses were performed according to the types of CHIs and presented with pooled data simultaneously. The heterogeneity was judged based on the *I^2^* value and *P* value. If the studies had non-significant heterogeneity within the studies or subgroups (*I^2^*<50%, *P*>0.1), we used a fixed effects model. If there was great heterogeneity within the studies or subgroups (*I^2^*>50%, *P*<0.1), we used the random effects model. If the data quantitative synthesis was not possible, we analyzed the available data qualitatively.

### Risk of Bias across trials

Funnel-plots were used to assess publication bias when the number of the included trials was more than 10.

### Additional analyses

To determine the robustness of results, sensitivity analyses were conducted based on the quality of trials 12, participants' number, treatment duration of CHIs, stage of cancer and publication year. Trial sequential analysis (TSA) was used to calculate the required information size (RIS) in the meta-analysis.

## Results

### Study Selection

**Figure 1:**
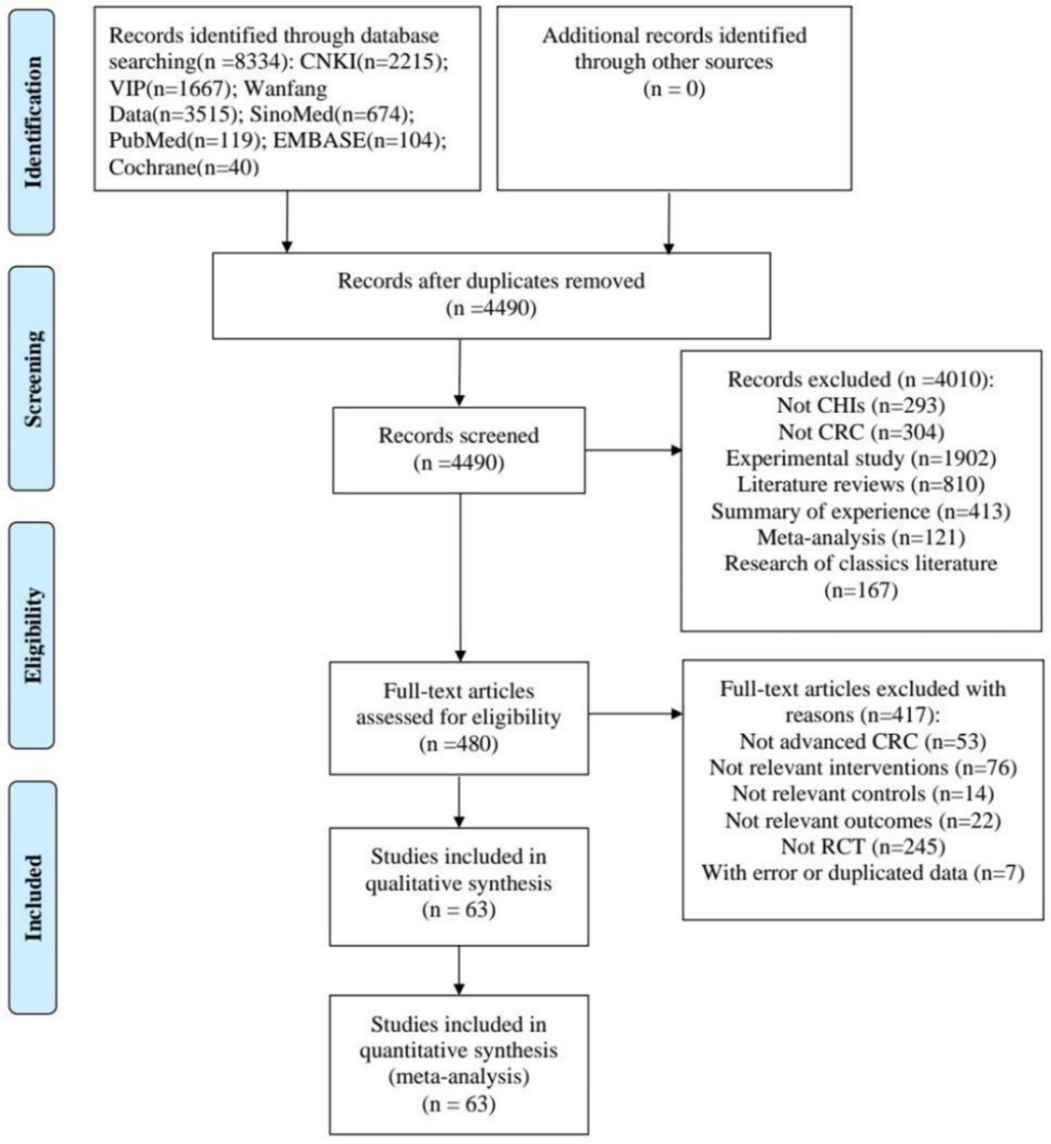
Flow diagram of the search for eligible studies.

The study selection process was described in **Figure 1.** A total of 8334 articles were identified from the initial literature search. After removing duplicates and irrelevant articles, 480 articles remained. Through reviewing the full texts of the remaining, a total of 63 papers 13-75 finally reached the criteria for entrance into the meta-analysis.

### Study Characteristics

63 RCTs recruiting 4733 patients were included. The baseline characteristics of the included trials were summarized in **Table 1.** All trials were conducted in China. All participants enrolled were patients with advanced CRC. There were 2394 and 2339 patients in the experimental and control groups, respectively. The number of participants in each RCT varied from 36 to 250. The numbers of studies included for 9 different CHIs were as follows: Compound Kushen injection (26 trials) 14, 17, 19, 21, 23, 27, 33, 34, 39-41, 43, 46, 47, 48, 51, 54, 55, 59-61, 65, 67, 68, 70, 71; Aidi injection (15 trials) 16, 18, 20, 22, 26, 28, 35, 37, 42, 44, 45, 53, 56, 64, 69; Shenqifuzheng injection (11 trials) 24, 25, 30, 31, 36, 38, 52, 58, 66, 73, 75; Kanglaite injection (3 trials) 57, 62, 63; Cinobufacini injection (3 trials) 13, 29, 50; Xiaoaiping injection (2 trials) 32, 72; Javanica oil emulsion injection (1 trial) 49; Astragalus injection (1 trial) 15; Lentinan injection (1 trial) 74.

### Quality evaluation

**Figure 2:**
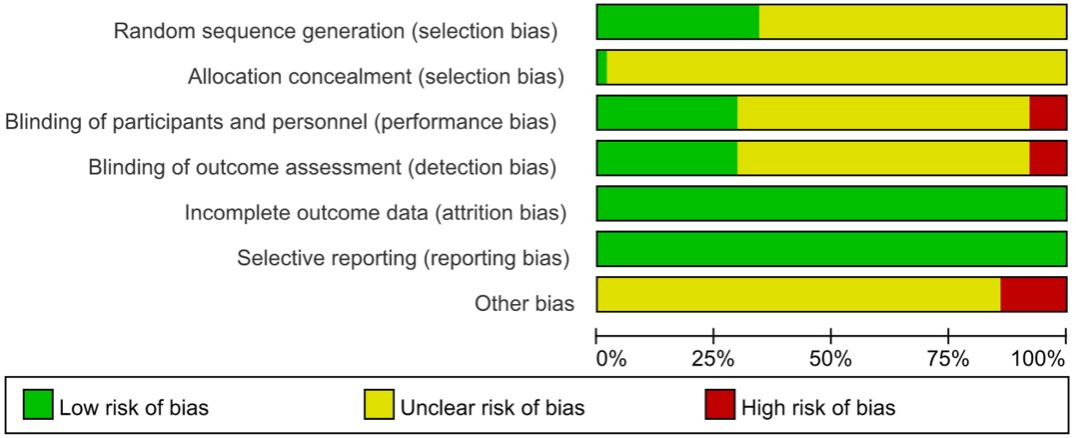
Risk of bias graph.

The results of the methodological evaluation were shown in **Figure 2.** With regard to random sequence generation, 22 studies 13, 17, 19, 22, 24, 26, 27, 32, 34, 36, 37, 47, 48, 50, 52, 56, 60, 62, 67, 69, 72, 73 were assessed as “low risk” because random number table and stratified randomization were adopted, the other studies did not report any randomization procedure, and were evaluated as “unclear”. Regarding allocation concealment, 1 trial 47 was evaluated as “low risk” because web-based central allocation was adopted, the risk of remaining RCTs were unclear.

Regarding blinding, since placebos were not mentioned in any of the studies, blinding was considered not performed in any of them. However, when the outcomes were ORR and DCR, it was considered that clinical judgements would not be influenced by lack of blinding because outcomes measured based on imaging. Therefore, 19 studies 17, 20, 22, 27, 31, 34, 37, 40, 46, 47, 48, 51, 56, 58, 62, 69, 70, 72, 73 that only reported ORR and DCR with clearly diagnostic criteria were evaluated as “low risk”. 40 studies 13-16, 18, 21, 23-25, 28-30, 32, 33, 35, 36, 38, 39, 41-45, 49, 50, 52-55, 57, 59, 61, 64-68, 71, 74, 75 in which subjective assessments were included in outcomes, making estimation of the influence of blinding on the study results difficult, were evaluated as “unclear”. 4 studies 19, 26, 60, 63 that only performed subjective assessments were evaluated as “high risk”, since blinding could have affected the study results.

The risk of incomplete outcome data was low as the reported data was consistence with the stated randomized numbers. The risk of selective reporting was low because the outcome results reported just as description in methods. Regarding other bias, 9 trials 13, 39, 45, 49, 53-55, 58, 62 took a range like “2-6” to limit the courses instead a definite figure or did not mention duration, which was a potential source of bias and were assessed as “high risk”, the other studies were not clear 76.

The results of the GRADE evaluation of studies which evaluated effectiveness and safety were presented in **Table 2.** All the reasons for downgrading are labeled 77.

### Effectiveness and safety

The findings of the meta-analyses were summarized in **Table 3,** and subgroup analyses conducted according to categories of CHIs were shown in **Table 4.**

#### Objective response rate (ORR)

**Figure 3:**
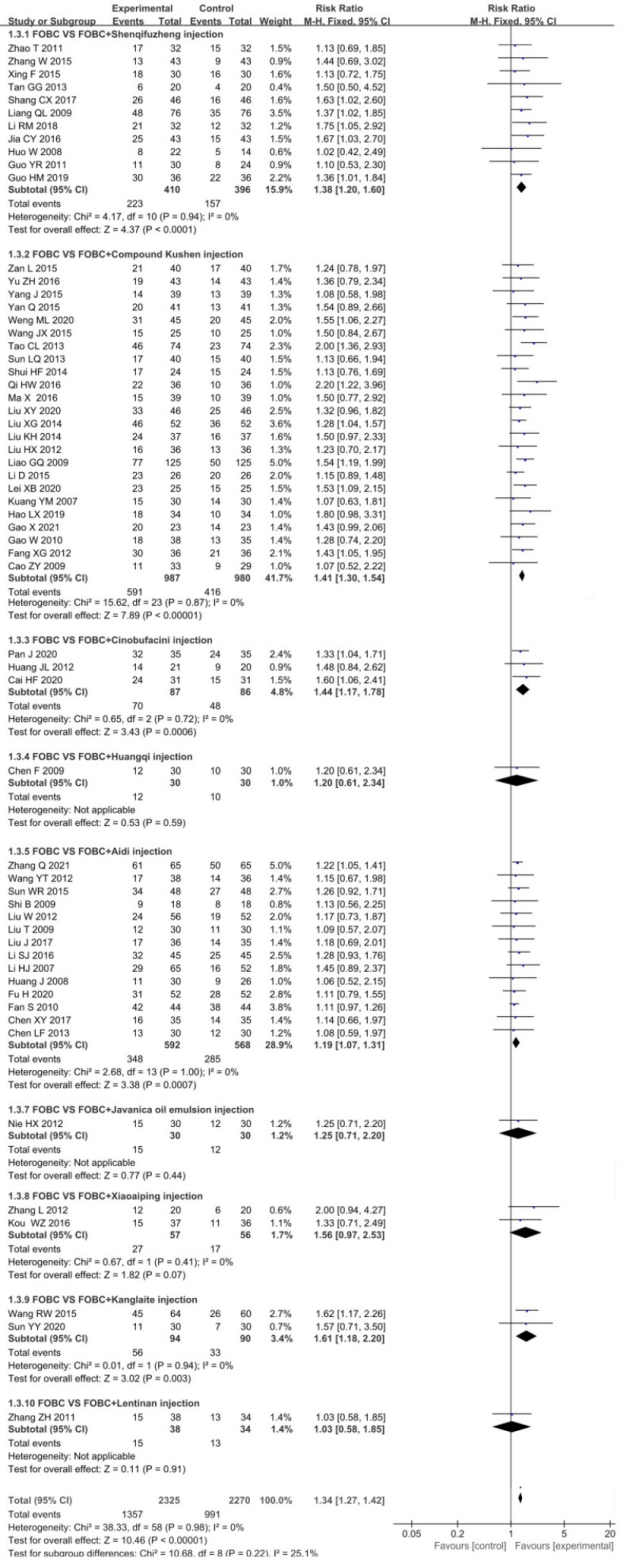
Forest plot of ORR in FOBC versus FOBC plus CHIs.

Data from 59 RCTs 13-18, 20-25, 27-48, 49-59, 61, 62, 64-75 with 9 types of CHIs contributed to the evidence for ORR. No statistical significant heterogeneity (*I^2^*=0%,* P*=0.98) was found and a fixed effect model was adopted. The results showed that the ORR was significantly enhanced in CHIs plus FOBC group when compared with FOBC group. (RR=1.34, 95%CI: 1.27-1.42, *P*<0.00001) (**Figure 3**). Subgroup analysis stratified by types of CHIs showed that the ORR was significantly enhanced in Compound Kushen injection subgroup (RR=1.41, 95% CI: 1.30-1.54, *P*<0.00001; *I^2^*=0%), Aidi injection subgroup (RR=1.19, 95% CI: 1.07-1.31, *P*= 0.0007; *I^2^*=0%), Shenqifuzheng injection subgroup (RR=1.38, 95% CI: 1.20-1.60, *P*<0.0001;* I^2^*=0%), Cinobufacini injection subgroup (RR=1.44, 95% CI: 1.17-1.78, *P*=0.0006; *I^2^*=0%), and Kanglaite injection subgroup (RR=1.61, 95% CI: 1.18-2.20,* P*=0.003;* I^2^*=0%), while showed no advantage in Xiaoaiping injection subgroup (RR=1.56, 95% CI: 0.97-2.53, *P*=0.07;* I^2^*=0%).

#### Disease control rate (DCR)

**Figure 4:**
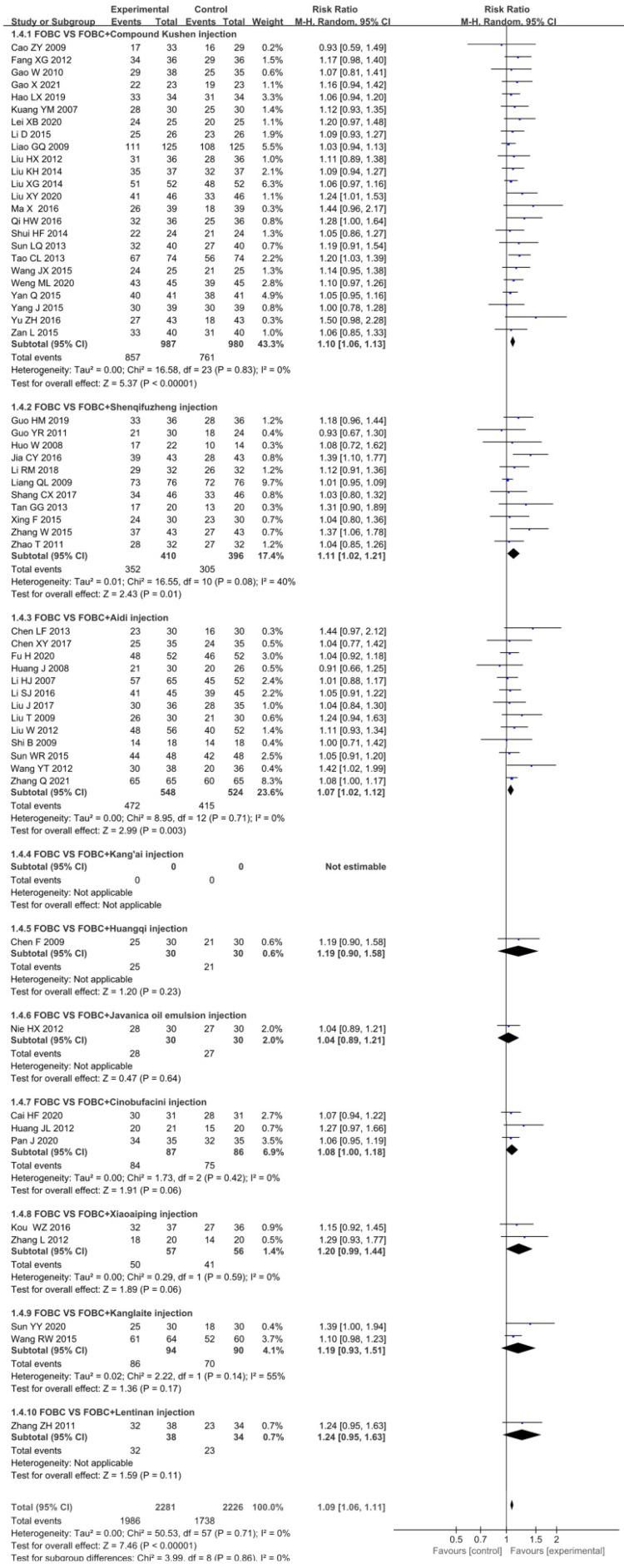
Forest plot of DCR in FOBC versus FOBC plus CHIs.

In total, 58 RCTs 13-18, 21-25, 27-48, 49-59, 61, 62, 64-75 with 9 CHIs contributed to the analysis of DCR with no significant heterogeneity *(I^2^*=0%,* P*=0.71). The results showed that the DCR was significantly enhanced in CHIs plus FOBC group than that in FOBC group. (RR=1.09, 95%CI: 1.06-1.11, *P* <0.00001) (**Figure 4**). Subgroup analysis indicated that DCR was enhanced in Compound Kushen injection subgroup (RR=1.10, 95% CI:1.06-1.13, *P*<0.00001; *I^2^*=0%), Shenqifuzheng injection subgroup (RR=1.11, 95% CI: 1.02-1.21,* P*=0.01;* I^2^*=40%), Aidi injection subgroup (RR=1.07, 95% CI: 1.02-1.12,* P*=0.003; *I^2^*=0%), while showed no advantage in Cinobufacini injection subgroup (RR=1.08, 95% CI: 1.00-1.18, *P*=0.06; *I^2^*=0%), Kanglaite injection subgroup (RR=1.19, 95% CI:0.93-1.51, *P*=0.17;* I^2^*=55%) and Xiaoaiping injection subgroup (RR=1.20, 95% CI: 0.99-1.44,* P*= 0.06; *I^2^*=0%).

#### Survival rate and Progression-free Survival (PFS)

**Figure 5:**
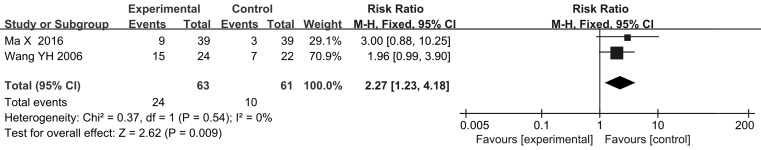
Forest plot of 1-year survival rate in FOBC versus FOBC plus CHIs.

There were 2 trials 47, 63 reported 1-year survival rate. The results indicated that the 1-year survival rate in CHIs plus FOBC group was higher than that in FOBC group (RR=2.27, 95% CI: 1.23-4.18, *P*=0.009) with low heterogeneity (*I^2^*=0%, *P*=0.54) (**Figure 5**).

2 RCTs 19, 69 reported progression-free survival (PFS), while because of the unextractable data and/or the diversity of survival outcomes in the included RCTs, meta-analysis was not possible for it 78.

#### Quality of life (QoL)

**Figure 6:**
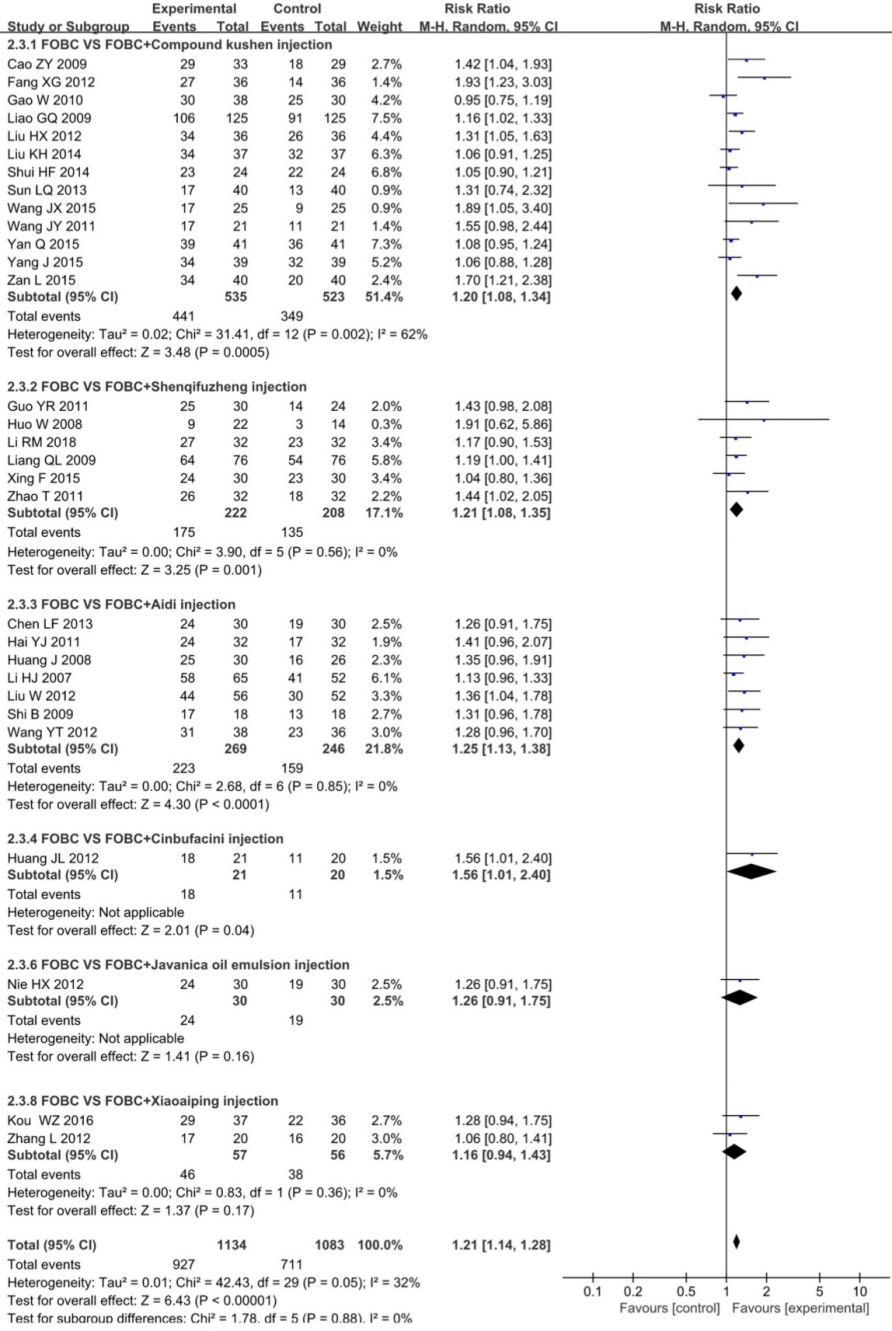
Forest plot of QoL in FOBC versus FOBC plus CHIs.

The data on the QoL was available for 30 trials 14, 16, 21, 23, 25, 26, 28-30, 32, 35, 36, 38, 39, 41, 43, 45, 49, 53-55, 60, 61, 64, 66-68, 71, 72, 75 involving 6 types of CHIs. The results showed that the QoL in CHIs plus FOBC group was significantly higher than that in FOBC group (RR=1.21, 95% CI: 1.14-1.28, *P*<0.00001) with low heterogeneity (*I^2^*=32%, *P*=0.05). Subgroup analysis indicated that QoL was significantly improved in Compound Kushen injection subgroup (RR=1.20, 95% CI: 1.08-1.34, *P*=0.0005; *I^2^*=62%), Aidi injection subgroup (RR=1.25, 95% CI: 1.13-1.38, *P*<0.0001; *I^2^*=0%) and Shenqifuzheng injection subgroup (RR=1.21, 95% CI: 1.08-1.35, *P*=0.001;* I^2^*=0%), while showed no advantage in Xiaoaiping injection subgroup (RR=1.16, 95% CI: 0.94-1.43, *P*=0.17;* I^2^*=0%) (**Figure 6**).

#### Leukopenia

**Figure 7:**
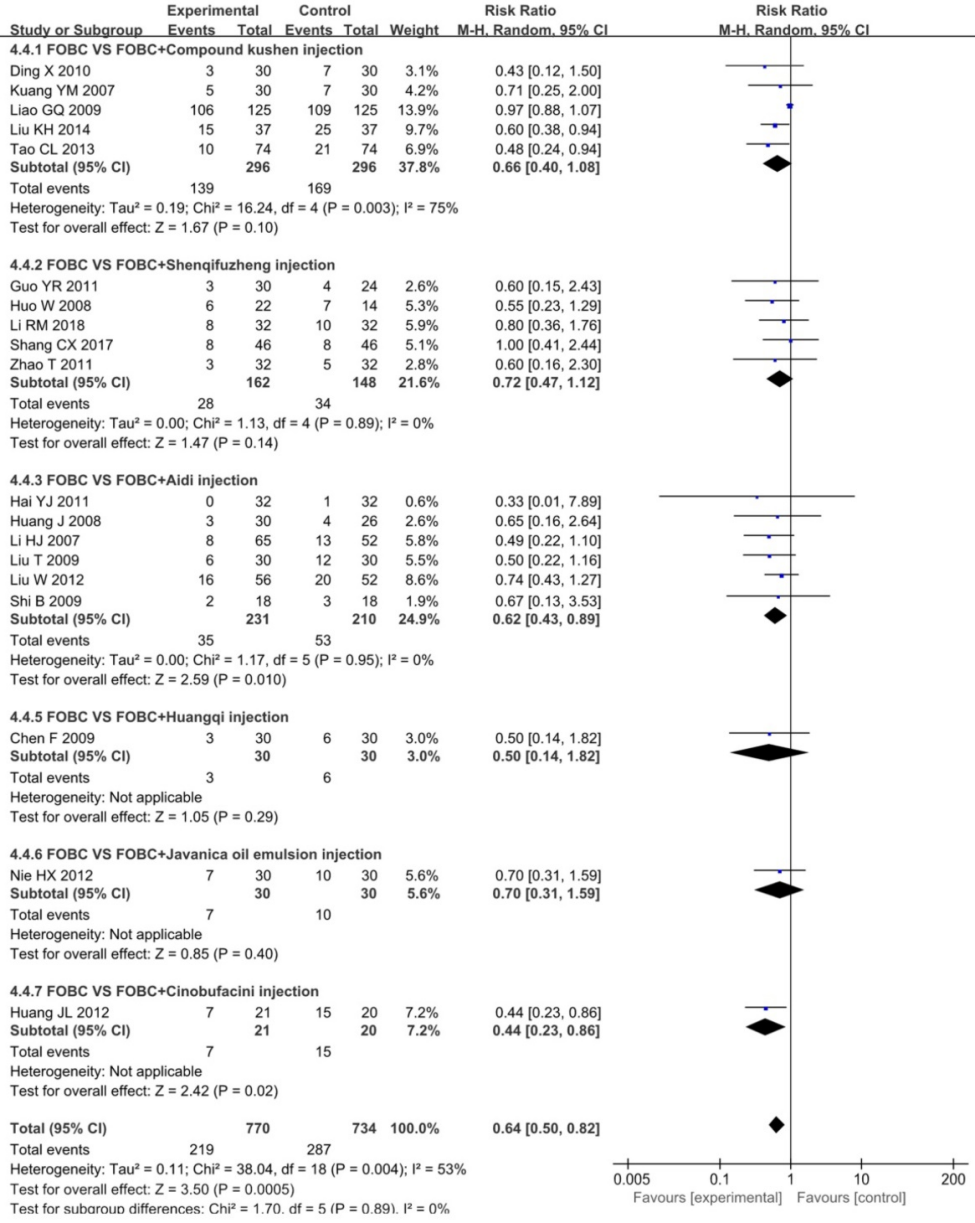
Forest plot of leukopenia in FOBC versus FOBC plus CHIs.

19 studies 15, 19, 25, 26, 28-30, 33, 35, 36, 39, 43-45, 49, 52, 53, 59, 75 with 6 types of CHIs reported the incidence of chemotherapy-induced leukopenia. **Figure 7** showed that the incidence of leukopenia in CHIs combined with FOBC group was lower than that in FOBC group (RR=0.64, 95% CI: 0.50-0.82, *P*=0.0005) with obvious heterogeneity (*I^2^*=53%, *P*=0.004). Subgroup analysis showed that the incidence of leukopenia was decreased in Aidi injection subgroup (RR=0.62, 95%CI: 0.43-0.89, *P*=0.01; *I^2^*=0%), but showed no advantage in Compound Kushen injection subgroup (RR=0.66, 95% CI: 0.40-1.08, *P*=0.10; *I^2^*=75%) and Shenqifuzheng injection subgroup (RR=0.72, 95% CI: 0.47-1.12, *P*=0.14;* I^2^*=0%).

#### Nausea and Vomiting

**Figure 8:**
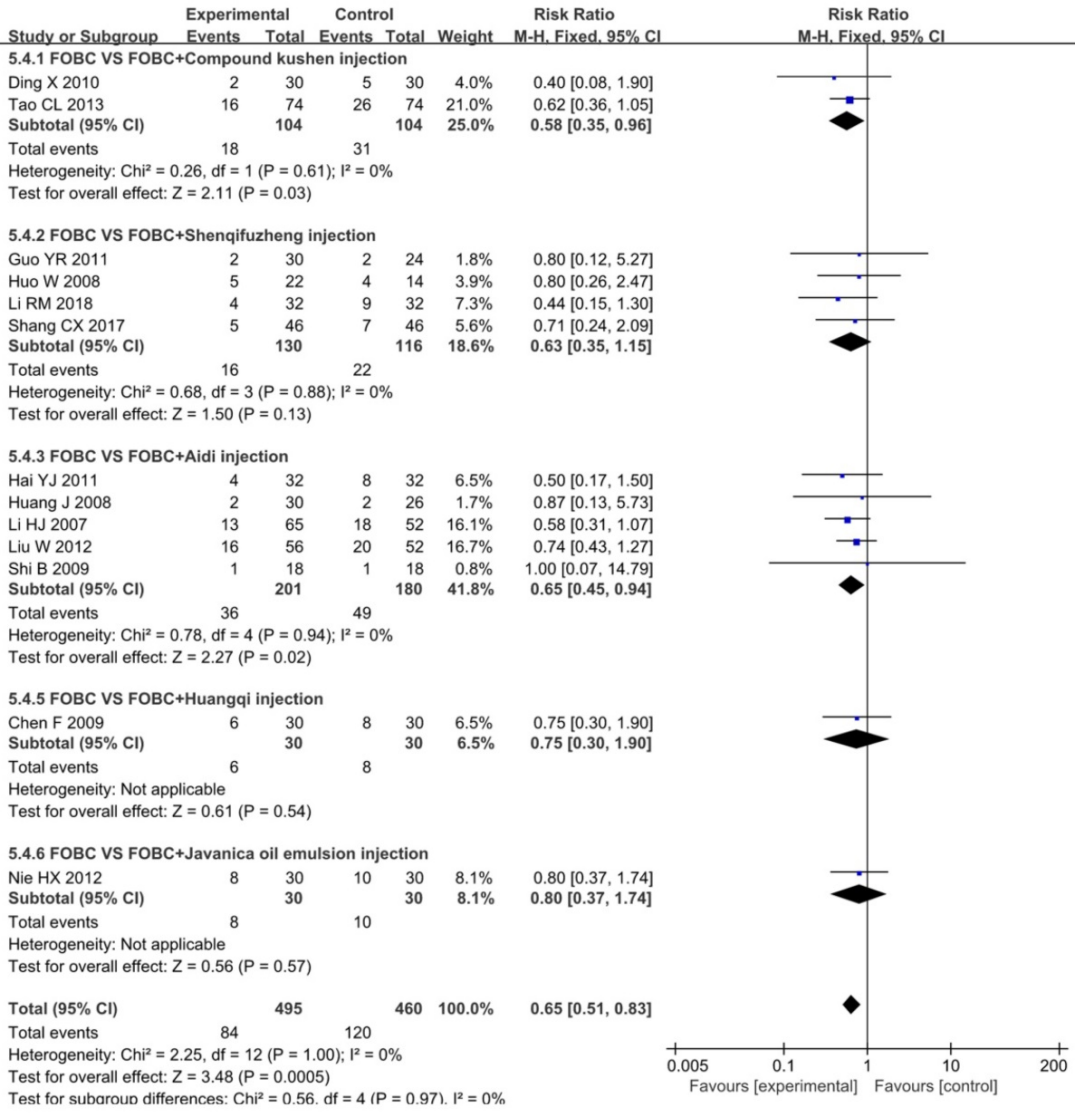
Forest plot of nausea and vomiting in FOBC versus FOBC plus CHIs.

A total of 13 studies 15, 19, 25, 26, 28, 30, 35, 36, 45, 49, 52, 53, 59 with 5 types of CHIs reported the data of nausea and vomiting, **Figure 8** exhibited that the incidence of nausea and vomiting in CHIs plus FOBC group was lower than that in FOBC alone group (RR=0.65, 95% CI: 0.51-0.83, *P*=0.0005. heterogeneity: *I^2^*=0%, *P*=1.00). Subgroup analysis showed that the incidence of nausea and vomiting was decreased in Aidi injection subgroup (RR=0.65, 95% CI: 0.45-0.94, *P*=0.02; *I^2^*=0%) and Compound Kushen injection subgroup (RR=0.58, 95% CI: 0.35-0.96, *P*=0.03; *I^2^*=0%), but showed no advantage in Shenqifuzheng injection subgroup (RR=0.63, 95% CI: 0.35-1.15, *P*=0.13; *I^2^*=0%).

#### Diarrhea

**Figure 9:**
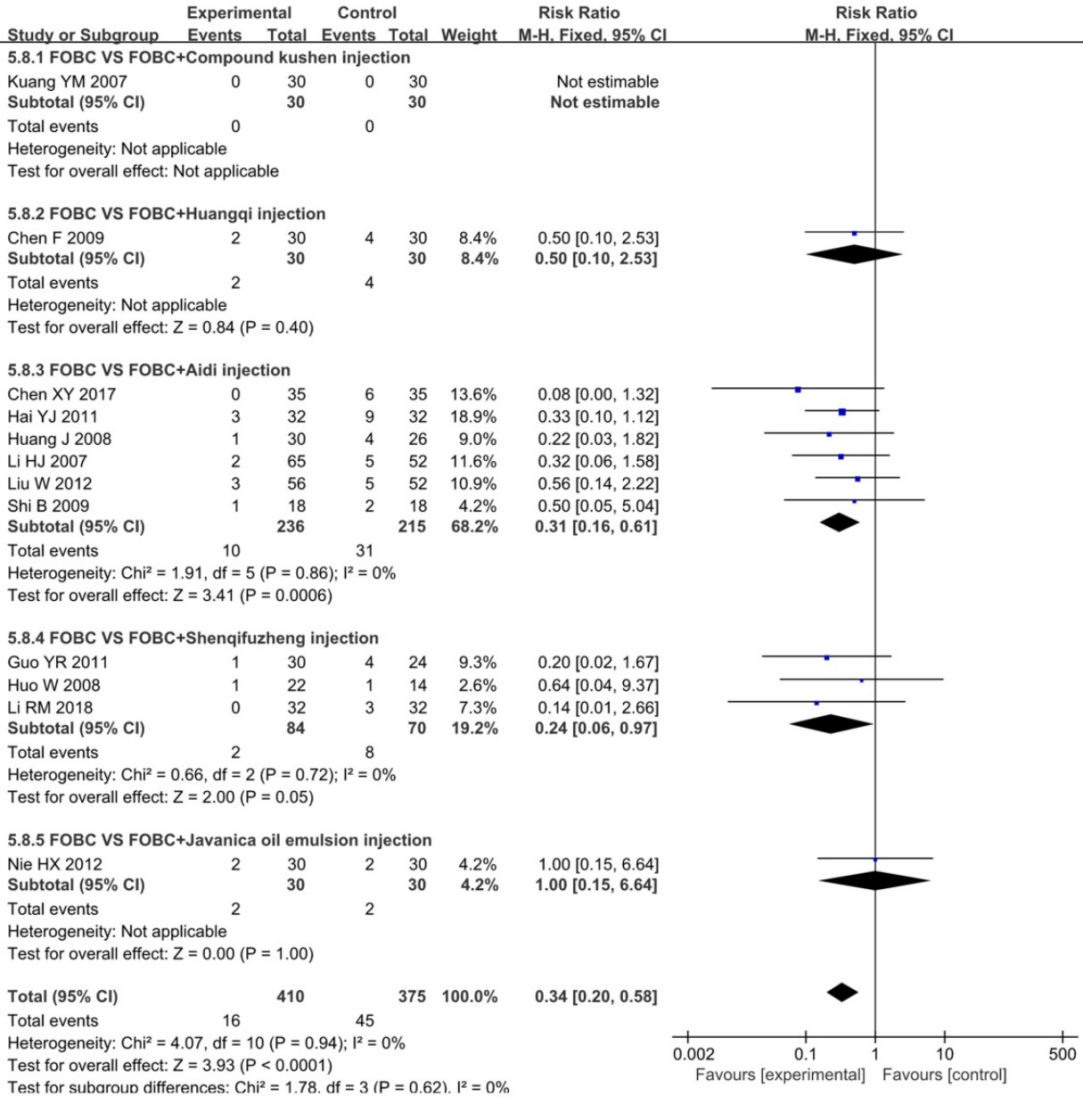
Forest plot of diarrhea in FOBC versus FOBC plus CHIs.

A total of 12 RCTs 15, 18, 25, 26, 28, 30, 33, 35, 36, 45, 49, 53 with 5 types of CHIs reported diarrhea, **Figure 9** showed that the incidence of diarrhea in CHIs plus FOBC group was lower than that in FOBC alone group (RR=0.34, 95% CI: 0.20-0.58, *P*<0.0001. heterogeneity: *I^2^*=0%, *P*=0.94). In subgroup analysis, the incidence of diarrhea was decreased in Shenqifuzheng injection subgroup (RR=0.24, 95% CI: 0.06-0.97, *P*=0.05; *I^2^*=0%) and Aidi injection subgroup (RR=0.31, 95% CI: 0.16-0.61, *P*=0.0006;* I^2^*=0%).

### Publication bias

**Figure 10:**
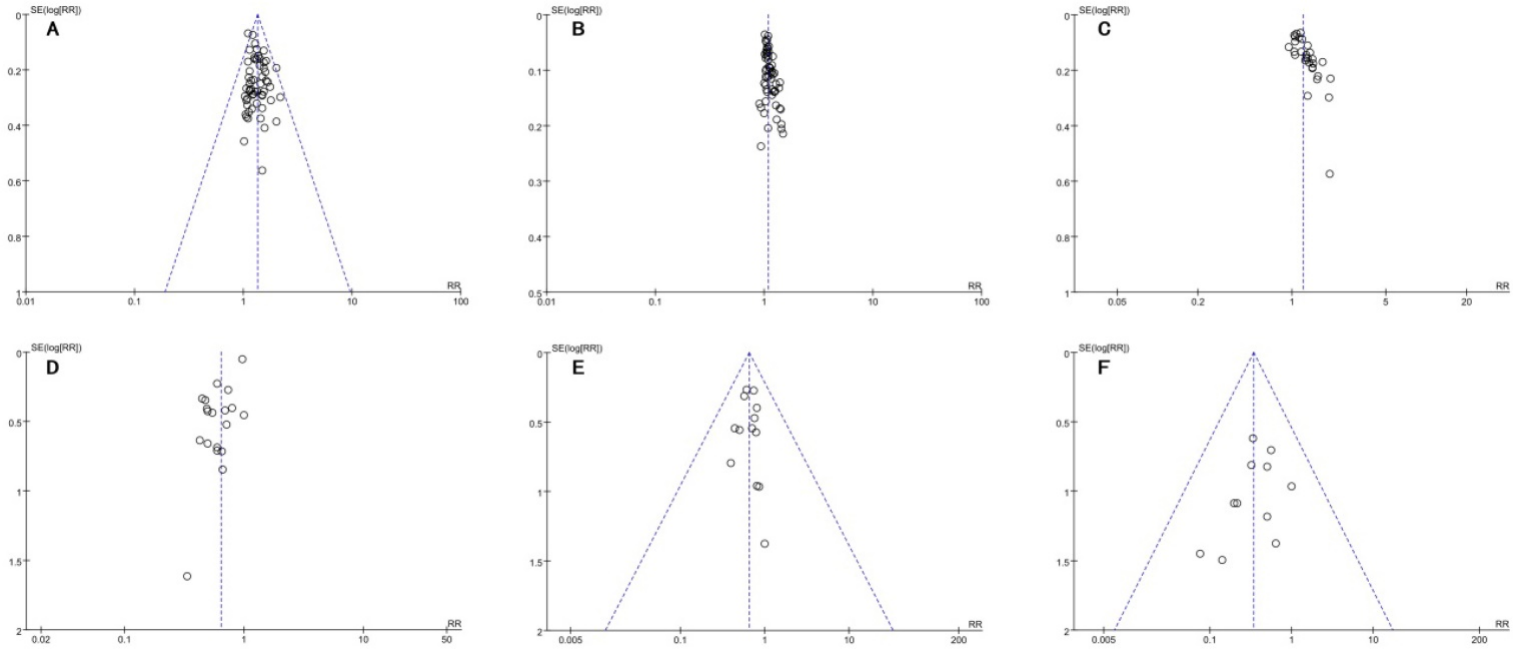
Funnel plots of outcomes: **A.** ORR; **B.** DCR; **C.** QoL; **D.** leukopenia; **E.** nausea and vomiting; **F.** diarrhea.

**Figure 10A-F** showed the funnel plots based on the data of the ORR, DCR, QoL, leukopenia, nausea and vomiting and diarrhea were asymmetrical, which indicated that publication bias might influence the results of the analysis.

### Additional analyses

Regarding ORR, the primary outcome, the pooled data showed that CHIs plus FOBC increased ORR significantly (RR=1.34, 95%CI: 1.27-1.42, *P*<0.00001). Similar increases were observed when the sensitivity analyses were performed based on the results of Cochrane Risk of Bias Tool (excluded 9 RCTs 13, 39, 45, 49, 53-55, 58, 62 of poor quality with at least one “high risk” domain in risk of bias assessment) (RR=1.33, 95% CI: 1.26-1.42, *P*<0.00001), participants number (only included 8 RCTs with ≥50 participants in each group) (RR=1.35, 95% CI: 1.22-1.49,* P*<0.00001), treatment duration of CHIs (only included 30 RCTs with ≥4 courses) (RR=1.30, 95% CI: 1.21-1.40 , P<0.00001), publication year (only included 21 studies published within 5 years) (RR=1.40, 95% CI: 1.29-1.53, P<0.00001) or stage (only included 6 studies enrolled patients with stage IV) (RR=1.59, 95% CI: 1.28-1.97, P<0.0001). It showed that the results of the primary outcome were robust before and after removing related trials (**Table 5**).

**Figure 11:**
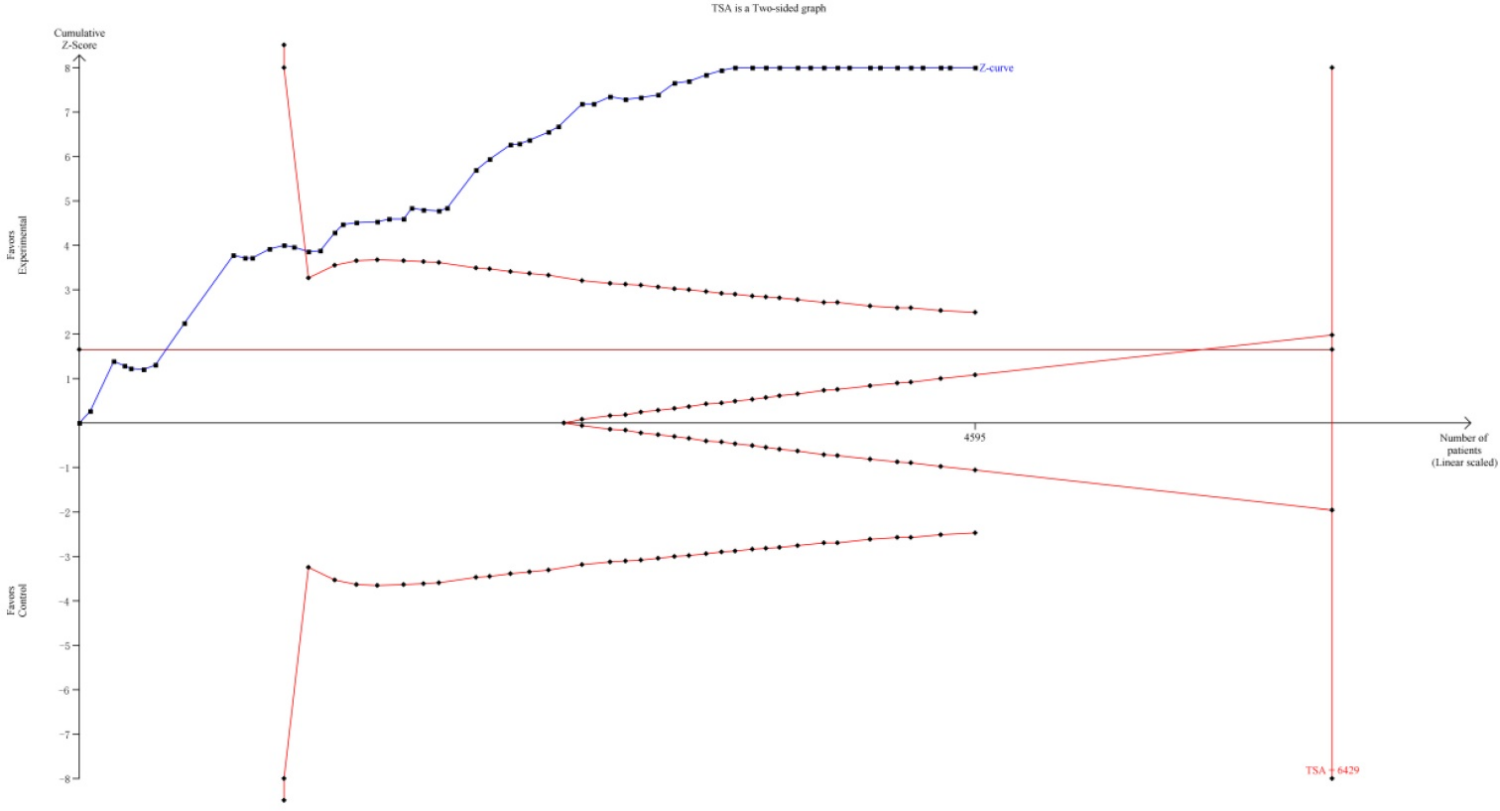
Trial sequential analysis (TSA) on ORR.

TSA was implemented to evaluate the required information size (RIS). As it showed in **Figure 11**, although the RIS has not been reached, the positive conclusion was obtained in advance as Z-curve had crossed the traditional boundary and TSA boundary. Therefore, it could be thought that CHIs combined with FOBC was significantly superior to FOBC alone in improving ORR, and the evidence was reliable 79.

## Discussion

To explore the effectiveness and safety of CHIs combined with FOBC in advanced CRC treatment, we conducted this meta-analysis to analyze the evidence in published RCTs. In general, the results of our study indicated that CHIs in conjunction with FOBC showed significant improvements in ORR, DCR, 1-year survival rate and QoL; and decreases in incidence of leukopenia, diarrhea and nausea and vomiting, while because of the unextractable data, whether PFS was improved remained unknown. In terms of methodology, the overall quality of the included studies could be considered moderate. GRADE assessments showed a low-quality of evidence. Most results showed low heterogeneity and good robustness.

Although FOBC regimen has been shown to prolong survival and reduce the advent of major complications in patients with advanced CRC 3, due to the lack of anti-tumor selective effects, it also has damaging effects on normal cells while suppressing tumor growth. Thus enhancing therapeutic effects and reducing adverse reactions became an urgent problem in current advanced CRC treatment 80, and CHIs proven to have those effects according to our results. As the products of the combination of TCM and modern science and technology, CHIs not only retain the characteristics of Chinese medicines under the guidance of Chinese medicine theory, but also obtain the advantages of modern chemical medicine like stable composition and fast onset. Compound Kushen injection is extracted from Kushen (Radix sophorae flavescentis) and Baituling (Rhizoma smilacis glabrae), it is extensively used for the treatment of malignant tumor such as liver cancer, lung cancer, and gastrointestinal cancer, and has been found to has the potiential to induce tumor cell differentiation and apoptosis and to inhibit tumor angiogenesis 81. The main active compounds of Aidi injection include cantharidin, astragalosides, ginsenosides, isofraxidin and syringin that are derived from Chinese herbs, various studies have shown that Aidi injection in relation to anti-tumor activity, immune regulatory action and adverse events relieving 82. Shenqifuzheng injection is composed of extracts from astragalus membranaceus and codonopsis pilosula, and was clinically indicated to improve body immunity and suppress tumor growth 83. Regarding effectiveness and safety of CHIs, our review indicated that some CHIs showed great beneficial impact on enhancing short-term effectiveness, improving 1-year survival rate and QoL, and reliving adverse effects.

To identify certain effective CHIs, we conducted subgroup analyses for all outcomes according to different types of CHIs as predefined. For the primary outcomes - ORR and DCR, 3 CHIs showed great advantages including Compound Kushen injection, Shenqifuzheng injection and Aidi injection. We also concerned that Xiaoaiping injection that extracted from Marsdenia tenacissima revealed no advantage in ORR, DCR, QoL and adverse reaction improvement, which suggested it might not recommended in the treatment of advanced CRC considering insufficient evidence.

There were some limitations in this study. First, all trials were conducted in China, which might lead to an unavoidable regional bias. Second, publication bias might exist on account of the asymmetrical funnel plots. Third, some studies lacked methodological details in randomization, allocation concealment and blinding, which might result in the emergence of bias and overestimation of effectiveness 84. Last, the study periods were generally short, and majority of the included trials did not report long-term endpoint outcomes such as overall survival (OS) and PFS that played a vital role in judging the therapeutic effects among patients with tumors. Given the limited quality and quantity of the included studies, more rigorous RCTs with high-quality methodology and long-term endpoint outcomes were needed to verify the beneficial role of CHIs combined with first-line chemotherapy in patients with advanced CRC.

## Conclusion

In conclusion, from the available evidence, CHIs could increase ORR and DCR, improve 1-year survival rate and QoL, and relieve leukopenia, nausea and vomiting and diarrhea when combined with FOBC in advanced CRC treatment. Meanwhile, considering the limitations, clinicians should choose carefully when applying the conclusions of this study.

## Supplementary Material

Supplementary search strategies.

## Figures and Tables

**Table 1:** Basic characteristics of the Included Studies

Study ID	N (T/C)	Age	TNM stages	Control group	Intervention group	CHIs dosage	CHIs Treatment	Interested outcomes
Cai HF 2020	31/31	T: 70.37C: 70.61	NR	5-FU+L-OHP+LV	CI + 5-FU+L-OHP+LV	20 mL/day	21 days/course, 2-4 courses	①②
Cao ZY 2009	33/29	T: 53.6C: 54.7	III-IV	5-FU+L-OHP+LV	CKS+ 5-FU+L-OHP+LV	15 mL/day	28 days/course, 2 courses	①②⑤⑥
Chen F 2009	30/30	T: 53.8±14.4C: 52.5±12.6	IV	5-FU+L-OHP+LV	AS + 5-FU+L-OHP+LV	60 mL/day	21 days/course, 3 courses	①②⑤⑥⑦
Chen LF 2013	30/30	T: 53.6C: 54.8	NR	5-FU+L-OHP+LV	AD+ 5-FU+L-OHP+LV	80 mL/day	10 days/course, 4 courses	①②⑤⑥⑦
Chen XY 2017	35/35	T: 49.36C: 42.66	NR	5-FU+L-OHP+LV	AD + 5-FU+L-OHP+LV	50 mL/day	21 days/course, 2 courses	①②⑦
Ding X 2010	30 /30	T: 64.5C: 63	NR	5-FU+L-OHP+LV	CKS + 5-FU+L-OHP+LV	20 mL/day	14 days/course, 4 courses	③⑥⑦
Fan S 2010	44/44	61	NR	5-FU+L-OHP+LV	AD+5-FU+L-OHP+LV	100 mL/day	14 days/course, 4 courses	①
Fang XG 2012	36/36	T: 49.8±4.1 C: 48.6±3.9	III-IV	5-FU+L-OHP+LV	CKS + 5-FU+L-OHP+LV	15 mL/day	14 days/course, 2 courses	①②⑤
Fu H 2020	52/52	T: 60.39C: 60.08	NR	CAP+L-OHP	AD+ CAP+L-OHP	50-100 mL/day	14 days/course, 2 courses	①②
Gao W 2010	38/35	NR	IV	5-FU+L-OHP+LV	CKS+ 5-FU+L-OHP+LV	20 mL/day	10 days/course, 4 courses	①②⑤
Guo HM 2019	36/36	T: 53.03C: 52.61	NR	5-FU+L-OHP+LV	SQFZ+5-FU+L-OHP+LV	250 mL/day	14 days/course, 4 courses	①②
Gao X 2021	23/23	T: 52.13±5.26C: 51.07±4.89	III-IV	5-FU+L-OHP+LV	CKS+ 5-FU+L-OHP+LV	15 ml/day	14 day/course, 4 courses	①②
Guo YR 2011	30/24	T: 65.4C: 66.5	III-IV	5-FU+L-OHP+LV	SQFZ+5-FU+L-OHP+LV	250 mL/day	14 days/course, 4 courses	①②⑤⑥⑦
Hai YJ 2011	32/32	56.4	III-IV	5-FU+L-OHP+LV	AD + 5-FU+L-OHP+LV	50 mL/day	10 days/course, 4 courses	⑥⑦
Hao LX 2019	34/34	76.26	NR	5-FU+L-OHP+LV	CKS+5-FU+L-OHP+LV	20 mL/day	14 days/course, 4 courses	①②
Huang J 2008	30/26	T: 65.4C: 66.5	III-IV	5-FU+L-OHP+LV	AD+ 5-FU+L-OHP+LV	50-100 mL/day	15 days/course, 2 courses	①②⑤⑥⑦
Huang JL 2012	21/20	T: 53.2C: 52.6	IV	5-FU+L-OHP+LV	CI+ 5-FU+L-OHP+LV	15-20 mL/day	14 days/course, 2 courses	①②⑤⑥
Huo W 2008	22/14	51	III-IV	5-FU+L-OHP+LV	SQFZ+5-FU+L-OHP+LV	250 mL/day	14 days/course, 4 courses	①②⑤⑥⑦
Jia CY 2016	43/43	T: 57.3C:56.7	NR	5-FU+L-OHP+LV	SQFZ+5-FU+L-OHP+LV	250 mL/day	14 days/course, 4 courses	①②
Kou WZ 2016	37/36	T: 56.8± 8.7C: 56.2 ± 8.5	III-IV	CAP+L-OHP	XAP+ CAP+L-OHP	40 mL/day	10 days/course, 2 courses	①②⑤
Kuang YM 2007	30/30	NR	NR	5-FU+L-OHP+LV	CKS+5-FU+L-OHP+LV	15 mL/day	10 days/course, 4 courses	①②⑥⑦
Li D 2015	26/26	T: 54.46±8.47C: 66.47±11.83	III-IV	5-FU+L-OHP+LV	CKS+5-FU+L-OHP+LV	15 mL/day	14 days/course, 4 courses	①②
Li HJ 2007	65/52	T: 58C: 59	NR	5-FU+L-OHP+LV	AD+ 5-FU+L-OHP+LV	60 mL/day	10 days/course, 4 courses	①②⑤⑥⑦
Li RM 2018	32 /32	T: 58C: 57	III-IV	CAP+L-OHP	SQFZ+ CAP+L-OHP	250 mL/day	7 days/course, 3 courses	①②⑤⑥⑦
Li SJ 2016	45/45	T: 54.82C: 54.67	NR	5-FU+L-OHP+LV	AD+ 5-FU+L-OHP+LV	100 mL/day	21 days/course, 2 courses	①②
Liang QL 2009	76 /76	NR	NR	5-FU+L-OHP+LV	SQFZ+5-FU+L-OHP+LV	250 mL/day	10 days/course, 2 courses	①②⑤
Liao GQ 2009	125/125	T: 58.6C:56.7	III-IV	5-FU+L-OHP+LV	CKS+5-FU+L-OHP+LV	20 mL/day	14 days/course	①②⑤⑥
Lei XB 2020	25/25	T: 57.21±1.64 C: 57.69±1.74	NR	5-FU+L-OHP+LV	CKS+ 5-FU+L-OHP+LV	15ml/day	7 days/course, 4 courses	①②
Liu HX 2012	36/36	T: 59.25±13.65C: 57.45±14.86	III-IV	5-FU+L-OHP+LV	CKS+ 5-FU+L-OHP+LV	40 mL/day	15 days/course, 3 courses+	①②⑤⑥
Liu J 2017	36/35	T: 65.3±3.2C: 64.8±3.1	NR	5-FU+L-OHP+LV	AD + 5-FU+L-OHP+LV	50 mL/day	21 days/course, 2 courses	①②
Liu KH 2014	37/37	T: 61C: 59	IIIb-IV	5-FU+L-OHP+LV	CKS+5-FU+L-OHP+LV	20 mL/day	14 days/course, 4 courses	①②⑤⑥
Liu T 2009	30/30	T: 63.2C:62.2	NR	5-FU+L-OHP+LV	AD+5-FU+L-OHP+LV	50 mL/day	21 days/course, 2 courses	①②⑥⑦
Liu W 2012	56/52	T:58.5C:60.2	NR	5-FU+L-OHP+LV	AD+ 5-FU+L-OHP+LV	50 mL/day	28 days/course, (2-6) courses	①②⑤⑥⑦
Liu XG 2014	52/52	T: 59.5±3.6C: 58.6±3.4	NR	5-FU+L-OHP+LV	CKS+ 5-FU+L-OHP+LV	20 mL/day	14 days/course, 6 courses	①②
Liu XY 2020	46/46	T: 53.81± 4.01 C: 54.09±3.93	IIIb-IV	CAP+L-OHP	CKS+CAP+L-OHP	15 mL/day	21 day/course, 4 courses	①②
Ma X 2016	39/39	T: 48.27±5.31C: 48.36±5.58	III-IV	5-FU+L-OHP+LV	CKS+ 5-FU+L-OHP+LV	20 mL/day	7 days/course, 8 courses	①②④
Nie HX 2012	30/30	T: 50C: 52	III-IV	5-FU+L-OHP+LV	JOE+ 5-FU+L-OHP+LV	30 mL/day	21 days/course, 4+ courses	①②
Pan J 2020	35/35	T: 60.2±2.3 C: 59.8±2.5	III-IV	CAP+L-OHP	CI+ CAP+L-OHP	15-20 mL/day	7 days/course, 3 courses	①②⑥⑦
Qi HW 2016	36/36	T: 52.88±7. 11C: 64.82±7. 54	IV	5-FU+L-OHP+LV	CKS+ 5-FU+L-OHP+LV	15 mL/day	14 days/course, 3 courses	①②
Shang CX 2017	46/46	T: 57.31C: 58.11	III-IV	5-FU +L-OHP	SQFZ+ 5-FU +L-OHP	250 mL/day	21 days/course, 4 courses	①②⑥⑦
Shi B 2009	18/18	52	NR	CAP+L-OHP	AD+ CAP+L-OHP	50-100 mL/day	14 days/course, 2+ courses	①②⑤⑥⑦
Shui HF 2014	24 /24	56	NR	CAP+L-OHP	CKS+ CAP+L-OHP	20 mL/day	10 days/course, (4-6) courses	①②⑤
Sun LQ 2013	40/40	T: 61C: 60	NR	5-FU+L-OHP+LV	CKS+ 5-FU+L-OHP+LV	20 mL/day	21-28 days/course, 2 courses	①②⑤
Sun WR 2015	48/48	T: 55.82C: 55.67	NR	5-FU+L-OHP+LV	AD+ 5-FU+L-OHP+LV	100 mL/day	21 days/course, 4 courses	①②
Sun YY 2020	30/30	T: 53.8±6.1C: 55.3±5.8	III-IV	5-FU+L-OHP+LV	KLT+5-FU+L-OHP+LV	200 mL/day	28 days	①②⑥⑦
Tan GG 2013	20/20	64	III-IV	CAP+L-OHP	SQFZ+ CAP+L-OHP	250 mL/day	14 days/course, 2+ courses	①②
Tao CL 2013	36/38	T: 60.1±7.9C: 60.4±8.9	IV	5-FU+L-OHP+LV	CKS+5-FU+L-OHP+LV	15 mL/day	14 days/course, 3 courses	①②⑤⑥⑦
Wang JX 2015	25/25	T: 58C: 60	IIIb-IV	5-FU+L-OHP+LV	CKS + 5-FU+L-OHP+LV	12 mL/day	14 days/course, 4 courses	①②⑤⑥
Wang JY 2011	21/21	T: 55.25C: 54.80	IV	5-FU+L-OHP+LV	CKS+ 5-FU+L-OHP+LV	12 mL/day	200 mL/course, 2-4 courses	⑤
Wang RW 2015	64/60	T: 51.9±3.1C: 50.5±3.3	III-IV	5-FU+L-OHP+LV	KLT+ 5-FU+L-OHP+LV	100mg/day	NR	①②⑤
Wang YH 2006	24/22	T: 57.1C: 58.2	IV	5-FU+L-OHP+LV	KLT+5-FU+L-OHP+LV	100 mL/day	21 days/course, 6 courses	④
Wang YT 2012	38/36	52	NR	5-FU+L-OHP+LV	AD+ 5-FU+L-OHP+LV	80 mL/day	10 days/course, 4 courses	①②⑤
Weng ML 2020	45/45	T: 51.07±5.21 C: 50.11±4.25	III-IV	5-FU+L-OHP+LV	CKS+ 5-FU+L-OHP+LV	20 mL/day	7 days/course, 4 courses	①②⑤⑥
Xing F 2015	30/30	T: 52C: 53	III-IV	5-FU+L-OHP+LV	SQFZ+5-FU+L-OHP+LV	250 mL/day	10 days/course, 4 courses	①②⑤
Yan Q 2015	41/41	T: 55.1C: 53.6	NR	5-FU+L-OHP+LV	CKS+ 5-FU+L-OHP+LV	20 mL/day	7 days/course, 4 courses	①②⑤
Yang J 2015	39/39	T: 55.1C: 53.8	IV	CAP+L-OHP	CKS+ CAP+L-OHP	12 mL/day	7 days/course, 4 courses	①②⑤
Yu ZH 2016	43/43	T:54.59C:4.85	NR	5-FU+L-OHP+LV	CKS+5-FU+L-OHP+LV	40 mL/day	15 days/course, 4 courses	①②
Zan L 2015	40/40	T: 52C: 51	III-IV	5-FU+L-OHP+LV	CKS+ 5-FU+L-OHP+LV	30 mL/day	7 days/course, 2 courses	①②⑤
Zhang L 2012	20/20	T: 59.24±20.37 C: 61.56±21.53	III-IV	5-FU+L-OHP+LV	XAP+ 5-FU+L-OHP+LV	1 mg/day	7 days/course, 2 courses	①②⑤
Zhang Q 2021	65/65	T: 59±10 C: 59±10	III-IV	5-FU+L-OHP+LV	AD+5-FU+L-OHP+LV	50ml/day	14 days/course, 4 courses	①②
Zhang W 2015	43/43	64.3	III-IV	CAP+L-OHP	SQFZ+ CAP+L-OHP	250 mL/day	14 days/course, 2 courses	①②
Zhang ZH 2011	38/34	T: 58 C: 59	NR	5-FU+L-OHP+LV	LE+ 5-FU+L-OHP+LV	1 mg/day	14 days/course, 8 courses	①②⑦
Zhao T 2011	32/32	NR	NR	5-FU+L-OHP+LV	SQFZ+5-FU+L-OHP+LV	250 mL/day	14 days/course, 2 courses	①②⑤⑥⑦

5-FU: 5-fluorouracil; AD: Aidi injection; AS: Astragalus injection; C: control group; CAP: Capecitabine; CI: Cinobufacini injection; CKS: Compound Kushen injection; JOE: Javanica oil emulsion injection; KA: Kangai injection; KLT: Kanglaite injection; LE: Lentinan injection; LV: leucovorin; NR: not reported, and the “NR” in the column of “TNM stages” means not reporting exact stages but mentioning “advanced”; SQFZ: Shenqifuzheng injection; T: treatment group; XAP: Xiaoaiping injection; ①: ORR; ②: DCR; ③: PFS; ④: survival rate; ⑤: quality of life; ⑥: leukopenia; ⑦: gastrointestinal side effects.

**Table 2:** The results of GRADE evaluation

Quality assessment	Numbers of RCTs	Risk of bias	Inconsistency	Indirectness	Imprecision	Publication bias	RR (95% CI)	Certainty of evidence
ORR	59	serious^a^	not serious (*I^2^=*0%*, P=*0.98)	not serious	not serious	strongly suspected^d^	1.34 (1.27-1.42)	LOW
DCR	58	serious^a^	not serious (*I^2^=*0%*, P*=0.71)	not serious	not serious	strongly suspected^d^	1.09 (1.06-1.11)	LOW
1-year survival rate	2	serious^a^	not serious (*I^2^=*0%*, P*=0.54)	not serious	very serious^c^	undetected	2.27 (1.23-4.18)	VERY LOW
Quality of life	30	very serious^a^	serious^b^ (*I^2^=*32%*, P*=0.05)	not serious	not serious	strongly suspected^d^	1.21 (1.14-1.28)	VERY LOW
Leukopenia	20	serious^a^	serious^b^ (*I^2^*=53%, *P*=0.004)	not serious	not serious	strongly suspected^d^	0.64 (0.50-0.82)	VERY LOW
Nausea and vomiting	13	serious^a^	not serious (*I^2^=*0%*, P=*1.00)	not serious	serious^c^	strongly suspected^d^	0.65 (0.51-0.83)	VERY LOW
Diarrhea	12	serious^a^	not serious (*I^2^=*0%*, P=*0.94)	not serious	Not serious	strongly suspected^d^	0.34 (0.20-0.58)	LOW

a Unclear description of the hidden methods of random sequence and random allocation. b Point estimates vary widely from study to study. c The number of studies was too small and the confidence interval was too wide to be accurate. d The funnel plots were asymmetrical, which indicated that publication bias might influence the results of the analysis.

**Table 3:** Summary of the Meta-analysis

Outcomes	Studies	Participants	Statistical methods	Pooled RRs (95% CI)	*P*	Heterogeneity	
						*I^2^*	*P_h_*
ORR	59	4595	FEM	1.34 (1.27-1.42)	<0.00001	0%	0.98
DCR	58	4507	REM	1.09 (1.06-1.11)	<0.00001	0%	0.71
1-year survival rate	2	124	FEM	2.27 (1.23-4.18)	0.009	0%	0.54
QoL	30	2217	REM	1.21 (1.14-1.28)	<0.00001	32%	0.05
Leukopenia	20	1504	REM	0.64 (0.50-0.82)	0.0005	53%	0.004
Nausea and vomiting	13	955	FEM	0.65 (0.51-0.83)	0.0005	0%	1.00
Diarrhea	12	785	FEM	0.34 (0.20-0.58)	<0.0001	0%	0.94

FEM: fixed-effects model; CI: confidence interval; RRs: risk ratios; REM: random-effects model.

**Table 4:** Subgroup analyses of all outcomes

Subgroups	Number of studies	Pooled RRs (95% CI)	*Z*	*P*	Heterogeneity	
					*I^2^*	*P_h_*
**ORR**						
Compound Kushen injection	24	1.41 (1.30, 1.54)	7.89	<0.00001	0%	0.87
Aidi injection	14	1.19 (1.07, 1.31)	3.38	0.0007	0%	1.00
Shenqifuzheng injection	11	1.38 (1.20, 1.60)	4.37	<0.0001	0%	0.94
Kanglaite injection	2	1.61 (1.18, 2.20)	3.02	0.003	0%	0.94
Cinobufacini injection	3	1.44 (1.17, 1.78)	3.43	0.0006	0%	0.72
Xiaoaiping injection	2	1.56 (0.97, 2.53)	1.82	0.07	0%	0.41
**DCR**						
Compound Kushen injection	24	1.10 (1.06, 1.13)	5.37	<0.00001	0%	0.83
Aidi injection	13	1.07 (1.02, 1.12)	2.99	0.003	0%	0.71
Shenqifuzheng injection	11	1.11 (1.02, 1.21)	2.43	0.01	40%	0.08
Kanglaite injection	2	1.19 (0.93, 1.51)	1.36	0.17	55%	0.14
Cinobufacini injection	3	1.08 (1.00, 1.18)	1.91	0.06	0%	0.59
Xiaoaiping injection	2	1.20 (0.99, 1.44)	1.89	0.06	0%	0.59
**QoL**						
Compound Kushen injection	13	1.20 (1.08, 1.34)	3.48	0.0005	62%	0.002
Aidi injection	7	1.25 (1.13, 1.38)	4.30	<0.0001	0%	0.85
Shenqifuzheng injection	6	1.21 (1.08, 1.35)	3.25	0.001	0%	0.56
Xiaoaiping injection	2	1.16 (0.94, 1.43)	1.37	0.17	0%	0.36
**Leukopenia**						
Compound Kushen injection	5	0.66 (0.40, 1.08)	1.67	0.10	75%	0.003
Aidi injection	6	0.62 (0.43, 0.89)	2.59	0.010	0%	0.95
Shenqifuzheng injection	5	0.72 (0.47, 1.12)	1.47	0.14	0%	0.89
**Nausea and vomiting**						
Compound Kushen injection	2	0.58 (0.35, 0.96)	2.11	0.03	0%	0.61
Aidi injection	5	0.65 (0.45, 0.94)	2.27	0.02	0%	0.94
Shenqifuzheng injection	4	0.63 (0.35, 1.15)	1.50	0.13	0%	0.88
**Diarrhea**						
Aidi injection	6	0.31 (0.16, 0.61)	3.41	0.0006	0%	0.86
Shenqifuzheng injection	3	0.24 (0.06, 0.97)	2.00	0.05	0%	0.72

CI: confidence interval; DCR: disease control rate; ORR: objective response rate; RRs: risk ratios; QoL: quality of life.

**Table 5:** Sensitivity analysis of objective response rate (ORR)

Types	Excluded trials (references)	Remaining trials	Statistical methods	RRs (95%CI)	*P*	*I2*
Quality of trials	9 13, 39, 45, 49, 53-55, 58, 62	50	FEM	1.33 (1.26, 1.42)	< 0.00001	0%
Participants number	51 13-18, 20, 21, 23-25, 27-34, 36, 37, 40-44, 47-59, 61, 64-68, 70-75	8	FEM	1.35(1.22, 1.49)	< 0.00001	0%
Treatment duration of CHIs	29^13^-^15^, ^18^, ^21^, ^22^, ^28^, ^29^, ^32^, ^36^-^39^, ^41^, ^42^, ^44^, ^45^, ^50^, ^51^, ^53^, ^55^, ^57^-^59^, ^62^, ^71^-^73^, ^75^	30	FEM	1.30 (1.21, 1.40)	< 0.00001	0%
Publication year	38 14-16, 20, 21, 23, 25, 28-30, 33-35, 38, 39, 41, 43-46, 49, 53-56, 58,59, 61, 62, 64, 66-68, 71-75	21	FEM	1.40 (1.29, 1.53)	< 0.00001	0%
Stage	53 13, 14, 16-18, 20-22, 24, 25, 27, 28, 30-50, 52-58, 61, 62, 64-67, 69-75	6	FEM	1.59 (1.28, 1.97)	< 0.0001	9%

FEM: fixed-effects model; RRs: risk ratios; CI: confidence interval.

## Abbreviations

CHIs: Chinese herbal injections
CI: Confidence Interval
CNKI: China National Knowledge Infrastructure
CR: complete response rates
CRC: colorectal cancer
DCR: disease control rate
FOBC: fluoropyrimidines and oxaliplatin-based chemotherapy
FUs: Fluoropyrimidines
GRADE: Grading of Recommendations, Assessment, Development and Evaluation
IARC: International Agency for Research on Cancer
INPLASY: International Platform of Registered Systematic Review and Meta-analysis Protocols
KPS: Karnofsky performance scale
NCI-CTCAE: National Cancer Institute Common Terminology Criteria for Adverse Events
NIH: National Cancer Institute
RR: risk ratio
ORR: objective response rate
OS: overall survival
PD: progressive disease
PR: partial response
PFS: progression-free survival
PRISMA: Preferred Reporting Items for Systematic Reviews and Meta-Analysis
QoL: quality of life
RCT: randomized controlled trial
RECIST: Response Evaluation Criteria in Solid Tumors
SD: stable disease
TCM: Traditional Chinese Medicine
TSA: Trial sequential analysis
WHO: World Health Organization
5-FU: 5-fluorouracil
