# Supplementary material for: Effectiveness and Safety of Chinese Herbal Injections Combined with Fluoropyrimidine and Oxaliplatin-based Chemotherapy for Advanced Colorectal Cancer: A Systematic Review and Meta-analysis of 63 Randomized Controlled Trials
Source: J Cancer. 2021 Oct 25;12(23):7237–54. doi: 10.7150/jca.60895 (PMC8558662; doi:10.7150/jca.60895)
Supplement: Supplementary file 1 — Supplementary search strategies. [file jcav12p7237s1.pdf]

File S1: Search Strategies

Searching words of Chinese herbal injections

Names of Chinese Herbal injections	English Searching Words	Chinese Searching Words
艾迪注射液	Aidi	艾迪注射液 OR 艾迪注射剂 OR 爱迪注射液 OR 爱迪注射剂 OR 注射用艾迪 OR 艾迪液
蟾酥注射液	Chansu OR Toad venom	佳素 OR 史君轻 OR 蟾毒康 OR 蟾酥注射液 OR 蟾酥注射剂
复方苦参注射液	Compound matrine OR Compound Kushen OR Fufangkushen	岩舒注射液 OR 岩舒 OR 复方苦参注射液 OR 复方苦参注射剂 OR 复方苦参
华蟾素注射液	Huachansu OR Cinobufacini	华蟾素注射液 OR 华蟾素注射剂 OR 华蟾素
康莱特注射液	Kanglaite	ZCE-3 静脉乳 OR 薏苡仁提取液 OR 注射薏苡仁油 OR 薏苡仁酯 OR 康莱特注射液 OR 康莱特注射剂
香菇多糖注射液	Xiangguduotang OR lentinan	香菇多糖注射液 OR 香菇多糖注射剂
消癌平注射液	Xiaoaiping OR Xiao-Ai-Ping OR Marsdenia Tenacissima	通关藤提取物 OR 通关藤注射液 OR 消癌平注射液 OR 消癌平注射剂 OR 消癌平
鸦胆子油乳注射液	Yadanziyouru OR Javanica oil emulsion OR Bmcea javanica	安体康注射液 OR 鸦胆子油乳注射液 OR 鸦胆子油乳注射剂 OR 鸦胆子油乳
参芪扶正注射液	Shenqifuzheng	参芪扶正注射液 OR 参芪扶正注射剂 OR 参芪扶正
康艾注射液	Kangai	康艾液 OR 康艾注射液 OR 康艾注射剂
参附注射液	Shenfu	参附注射液 OR 参附注射剂 OR 注射用参附

黄芪注射液	Huangqi OR Astragalus	黄芪注射液 OR 黄芪注射剂 OR 注射用黄芪冻干粉
香菇多糖注射液	Xiangguduotang OR Lentinan	力提能 OR 天地欣 OR 香菇多糖注射液 OR 香菇多糖注射剂 OR 注射用香菇多糖
参麦注射液	Shenmai	参麦注射液 OR 参麦注射剂 OR 注射用参麦
榄香烯注射液	Lanxiangxi OR Elemene	榄香烯注射液 OR 榄香注射液 OR β-榄香烯注射液 OR 榄香烯脂质体注射液 OR 榄香烯乳注射液

Search strategy of CNKI

((SU=中药注射剂 OR SU=中药注射液 OR SU=艾迪注射液 OR SU=艾迪注射剂 OR SU=爱迪注射液 OR SU=爱迪注射剂 OR SU=注射用艾迪 OR SU=艾迪液 OR SU=佳素 OR SU=史君轻 OR SU=蟾毒康 OR SU=蟾酥注射液 OR SU=蟾酥注射剂 OR SU=岩舒注射液 OR SU=岩舒 OR SU=复方苦参注射液 OR SU=复方苦参注射剂 OR SU=复方苦参 OR SU=华蟾素注射液 OR SU=华蟾素注射剂 OR SU=华蟾素 OR SU=ZCE-3 静脉乳 OR SU=薏苡仁提取液 OR SU=注射薏苡仁油 OR SU=薏苡仁酯 OR SU=康莱特注射液 OR SU=康莱特注射剂 OR SU=香菇多糖注射液 OR SU=香菇多糖注射剂 OR SU=通关藤提取物 OR SU=通关藤注射液 OR SU=消癌平注射液 OR SU=消癌平注射剂 OR SU=消癌平 OR SU=安体康注射液 OR SU=鸦胆子油乳注射液 OR SU=鸦胆子油乳注射剂 OR SU=鸦胆子油乳 OR SU=参芪扶正注射液 OR SU=参芪扶正注射剂 OR SU=参芪扶正 OR SU=康艾液 OR SU=康艾注射液 OR SU=康艾注射剂 OR SU=参附注射液 OR SU=参附注射剂 OR SU=注射用参附 OR SU=黄芪注射液 OR SU=黄芪注射剂 OR SU=注射用黄芪冻干粉 OR SU=力提能 OR SU=天地欣 OR SU=香菇多糖注射液 OR SU=香菇多糖注射剂 OR SU=注射用香菇多糖 OR SU=参麦注射液 OR SU=参麦注射剂 OR SU=注射用参麦 OR SU=榄香烯注射液 OR SU=榄香注射液 OR SU=β-榄香烯注射液 OR SU=榄香烯脂质体注射液 OR SU=榄香烯乳注射液) AND (SU=结肠癌 OR SU=直肠癌 OR SU=结直肠癌 OR SU=结肠肿瘤 OR SU=直肠肿瘤 OR SU=结直肠肿瘤 OR SU=大肠癌 OR SU=大肠肿瘤 OR SU=肠癌 OR SU=肠肿瘤)) NOT (TI=鼠 OR TI=兔)

Search strategy of VIP

((M=中药注射剂 OR M=中药注射液 OR M=艾迪注射液 OR M=艾迪注射剂 OR M=爱迪注射液 OR M=爱迪注射剂 OR M=注射用艾迪 OR M=艾迪液 OR M=佳素 OR M=史君轻 OR M=蟾毒康 OR M=蟾酥注射液 OR M=蟾酥注射剂 OR M=岩舒注射液 OR M=岩舒 OR M=复方苦参注射液 OR M=复方苦参注射剂 OR M=复方苦参 OR M=华蟾素注射液 OR M=华蟾素注射剂 OR M=华蟾素 OR M=ZCE-3 静脉乳 OR M=薏苡仁提取液 OR M=注射薏苡仁油 OR M=薏苡仁酯 OR M=康莱特注射液 OR M=康莱特注射剂 OR M=香菇多糖注射液 OR M=香菇多糖注射剂 OR M=通关藤提取物 OR M=通关藤注射液 OR M=消癌平注射液 OR M=消癌平注射剂 OR M=消癌平 OR M=安体康注射液 OR M=鸦胆子油乳注射液 OR M=鸦胆子油乳注射剂 OR M=鸦胆子油乳 OR M=参芪扶正注射液 OR M=参芪扶正注射剂 OR M=参芪扶正 OR M=康艾液 OR M=康艾注射液 OR M=康艾注射剂 OR M=参附注射液 OR M=参附注射剂 OR M=注射用参附 OR M=黄芪注射液 OR M=黄芪注射剂 OR M=注射用黄芪冻干粉 OR M=力提能 OR M=天地欣 OR M=香菇多糖注射液 OR M=香菇多糖注射剂 OR M=注射用香菇多糖 OR M=参麦注射液 OR M=参麦注射剂 OR M=注射用参麦 OR M=榄香烯注射液 OR M=榄香注射液 OR M=β-榄香烯注射液 OR M= 榄香烯脂质体注射液 OR M=榄香烯乳注射液) AND (M=结肠癌 OR M=直肠癌 OR M=结直肠癌 OR M=结肠肿瘤 OR M=直肠肿瘤 OR M=结直肠肿瘤 OR M=大肠癌 OR M=大肠肿瘤 OR M=肠癌 OR M=肠肿瘤)) NOT (T=鼠 OR T=兔)

Search strategy of Wanfang data

((主题:(中药注射剂+中药注射液+艾迪注射液+艾迪注射剂+爱迪注射液+爱迪注射剂+注射用艾迪+艾迪液+佳素+史君轻+蟾毒康+蟾酥注射液+蟾酥注射剂+岩舒注射液+岩舒+复方苦参注射液+复方苦参注射剂+复方苦参+华蟾素注射液+华蟾素注射剂+华蟾素+ZCE-3 静脉乳+薏苡仁提取液+注射薏苡仁油+薏苡仁酯+康莱特注射液+康莱特注射剂+香菇多糖注射液+香菇多糖注射剂+通关藤提取物+通关藤注射液+消癌平注射液+消癌平注射剂+消癌平+安体康注射液+鸦胆子油乳注射液+鸦胆子油乳注射剂+鸦胆子油乳+参芪扶正注射液+参芪扶正

注射剂+参芪扶正+康艾液+康艾注射液+康艾注射剂+参附注射液+参附注射剂+注射用参附+黄芪注射液+黄芪注射剂+注射用黄芪冻干粉+力提能+天地欣+香菇多糖注射液+香菇多糖注射剂+注射用香菇多糖+参麦注射液+参麦注射剂+注射用参麦+榄香烯注射液+榄香注射液+β-榄香烯注射液+ 榄香烯脂质体注射液+榄香烯乳注射液))* (主题:(结肠癌+直肠癌+结直肠癌+结肠肿瘤+直肠肿瘤+结直肠肿瘤+大肠癌+大肠肿瘤+肠癌+肠肿瘤))) not (题名:(兔)+(鼠))

Search strategy of SinoMed

((("结肠肿瘤"[不加权:扩展] OR "乙状结肠肿瘤"[不加权:扩展]) OR "直肠肿瘤"[不加权:扩展] OR "结直肠肿瘤, 遗传性非息肉性"[不加权:扩展]) OR "结直肠肿瘤"[不加权:扩展] OR "*结肠癌"[常用字段:智能] OR "*直肠癌"[常用字段:智能] OR "结直肠癌"[常用字段:智能] OR "大肠癌"[常用字段:智能] OR "肠癌"[常用字段:智能] OR "大肠肿瘤"[常用字段:智能] OR "肠肿瘤"[常用字段:智能]) AND ("中药注射剂"[常用字段:智能] OR "中药注射液"[常用字段:智能] OR "艾迪注射液"[常用字段:智能] OR "艾迪注射剂"[常用字段:智能] OR "爱迪注射液"[常用字段:智能] OR "爱迪注射剂"[常用字段:智能] OR "注射用艾迪"[常用字段:智能] OR "艾迪液"[常用字段:智能] OR "佳素"[常用字段:智能] OR "史君轻"[常用字段:智能] OR "蟾毒康"[常用字段:智能] OR "蟾酥注射液"[常用字段:智能] OR "蟾酥注射剂"[常用字段:智能] OR "岩舒注射液"[常用字段:智能] OR "岩舒"[常用字段:智能] OR "复方苦参注射液"[常用字段:智能] OR "复方苦参注射剂"[常用字段:智能] OR "复方苦参"[常用字段:智能] OR "华蟾素注射液"[常用字段:智能] OR "华蟾素注射剂"[常用字段:智能] OR "华蟾素"[常用字段:智能] OR "ZCE-3 静脉乳"[常用字段:智能] OR "薏苡仁提取液"[常用字段:智能] OR "注射薏苡仁油"[常用字段:智能] OR "薏苡仁酯"[常用字段:智能] OR "康莱特注射液"[常用字段:智能] OR "康莱特注射剂"[常用字段:智能] OR "香菇多糖注射液"[常用字段:智能] OR "香菇多糖注射剂"[常用字段:智能] OR "通关藤提取物"[常用字段:智能] OR "通关藤注射液"[常用字段:智能] OR "消癌平注射液"[常用字段:智能] OR "消癌平注射剂"[常用字段:智能] OR "消癌平"[常用字段:智能] OR "安体康注射液"[常用字段:智能] OR "鸦胆子油乳注射液"[常用字段:智能] OR "鸦胆子油乳注射剂"[常用字段:智能] OR "鸦胆子油乳"[常用字段:智能] OR "参芪扶正注射液"[常用字段:智能] OR "参芪扶正注射剂"[常用字段:智能] OR "参芪扶正"[常用字段:智能] OR "康艾液"[常用字段:智能] OR "康艾注射液"[常用字段:智能] OR "康艾注射剂"[常用字段:智能] OR "参附注射液"[常用字段:智能] OR "参附注射剂"[常用字段:智能] OR "注射用参附"[常用字段:智能] OR "黄芪注射液"[常用字段:智能] OR "黄芪注射剂"[常用字段:智能] OR "注射用黄芪冻干粉"[常用字段:智能] OR "力提能"[常用字段:智能] OR "天地欣"[常用字段:智能] OR "香菇多糖注射液"[常用字段:智能] OR "香菇多糖注射剂"[常用字段:智能] OR "注射用香菇多糖"[常用字段:智能] OR "参麦注射液"[常用字段:智能] OR "参麦注射剂"[常用字段:

智能] OR "注射用参麦"[常用字段:智能] OR "榄香烯注射液"[常用字段:智能] OR "榄香注射液"[常用字段:智能] OR "β-榄香烯注射液"[常用字段:智能] OR " 榄香烯脂质体注射液"[常用字段:智能] OR "榄香烯乳注射液)AND (讲座[文献类型] OR 译文[文献类型] OR 病例报告[文献类型] OR 临床试验[文献类型] OR 随机对照试验[文献类型] OR 多中心研究[文献类型]) AND (人类[特征词])

Search strategy of Pubmed

#1	(Colorectal Neoplasms[MeSH] OR Colonic Neoplasms[MeSH]) OR Rectal Neoplasms[MeSH]
#2	(tumor*[tiab] OR carcinoma*[tiab] OR neoplasm*[tiab] OR cancer*[tiab]) AND (coloretal[tiab] OR colon[tiab] OR colonic[tiab] OR rectal[tiab] OR retum[tiab])
#3	#1 OR #2
#4	Chinese herbal injection*[tiab] OR Chinese medicine injection[tiab] OR injection of TCM[tiab] OR Shenqifuzheng[tiab] OR Kanglaite[tiab] OR Compound Kushen[tiab] OR Fufangkushen[tiab] OR Compound matrine[tiab] OR Aidi[tiab] OR Cinobufotalin injection[tiab] OR Huachansu[tiab] OR Xiaoaiping[tiab] OR Xiao-Ai-Ping[tiab] OR Marsdenia Tenacissima[tiab] OR Elemene[tiab] OR Lanxiangxi[tiab] OR Xiangguduotang[tiab] OR lentinan[tiab] OR javanica oil emulsion[tiab] OR Bmcea javanica[tiab] OR Yadanziyouru[tiab] OR kang'ai[tiab] OR kangai[tiab] OR kang-ai[tiab] OR Huangqi[tiab] OR Astragalus[tiab] OR Shenfu[tiab] OR Shenmai[tiab]
#5	#3 AND #4

Search strategy of Embase

#1	'rectum tumor'/exp OR 'colon tumor'/exp
#2	(tumor*:ab,kw,ti OR carcinoma*:ab,kw,ti OR neoplasm*:ab,kw,ti OR cancer*:ab,kw,ti) AND (coloretal:ab,kw,ti OR colon:ab,kw,ti OR colonic:ab,kw,ti OR rectal:ab,kw,ti OR retum:ab,kw,ti)
#3	#1 OR #2

#4	'Chinese herbal injection*':ab,kw,ti OR 'Chinese medicine injection':ab,kw,ti OR 'injection of TCM':ab,kw,ti OR 'Shenqifuzheng':ab,kw,ti OR 'Kanglaite':ab,kw,ti OR 'Compound Kushen':ab,kw,ti OR 'Fufangkushen':ab,kw,ti OR 'Compound matrine':ab,kw,ti OR 'Aidi':ab,kw,ti OR 'Cinobufotalin injection':ab,kw,ti OR 'Huachansu':ab,kw,ti OR 'Xiaoaiping':ab,kw,ti OR 'Xiao-Ai-Ping':ab,kw,ti OR 'Marsdenia Tenacissima':ab,kw,ti OR 'Elemene':ab,kw,ti OR 'Lanxiangxi':ab,kw,ti OR 'Xiangguduotang':ab,kw,ti OR 'lentinan':ab,kw,ti OR 'javanica oil emulsion':ab,kw,ti OR 'Bmcea javanica':ab,kw,ti OR 'Yadanziyouru':ab,kw,ti OR 'kang'ai':ab,kw,ti OR 'kangai':ab,kw,ti OR 'kang-ai':ab,kw,ti OR 'Huangqi':ab,kw,ti OR 'Astragalus':ab,kw,ti OR 'Shenfu':ab,kw,ti OR 'Shenmai':ab,kw,ti
#5	#3 AND #4

Search strategy of Cochrane library

#1 MeSH descriptor: [Colorectal Neoplasms] explode all trees

#2 MeSH descriptor: [Colonic Neoplasms] explode all trees

#3 MeSH descriptor: [Rectal Neoplasms] explode all trees

#4 ((tumor* OR carcinoma* OR neoplasm* OR cancer*) AND (coloretal OR colon OR colonic OR rectal OR retum)):ti,ab,kw

#5 #1 or #2 or #3 or #4 or #5

#6 Chinese herbal injection*:ti,ab,kw or Chinese medicine injection:ti,ab,kw or injection of TCM:ti,ab,kw or Shenqifuzheng:ti,ab,kw or Kanglaite:ti,ab,kw or Compound Kushen:ti,ab,kw or Fufangkushen:ti,ab,kw or Compound matrine:ti,ab,kw or Aidi:ti,ab,kw or Cinobufotalin injection:ti,ab,kw or Huachansu:ti,ab,kw or Xiaoaiping:ti,ab,kw or Xiao-Ai-Ping:ti,ab,kw or Marsdenia Tenacissima:ti,ab,kw or Elemene:ti,ab,kw or Lanxiangxi:ti,ab,kw or Xiangguduotang:ti,ab,kw or lentinan:ti,ab,kw or javanica oil emulsion:ti,ab,kw or Bmcea javanica:ti,ab,kw or Yadanziyouru:ti,ab,kw or kang'ai:ti,ab,kw or kangai:ti,ab,kw or kang-ai:ti,ab,kw or Huangqi:ti,ab,kw or Astragalus:ti,ab,kw or Shenfu:ti,ab,kw or Shenmai:ti,ab,kw

#7 #5 and #6
